# A Rare Case of Extrinsic Stenosis of Dural Sinus

**DOI:** 10.5334/jbsr.2895

**Published:** 2022-09-30

**Authors:** Axel Boyer, Louis Deprez

**Affiliations:** 1 CHU de Liège, BE

**Keywords:** dural sinus stenosis, increased intracranial pressure

## Abstract

**Teaching Point:** Dural sinus stenosis are a rare cause of increased intracranial pressure and can be treated in some cases by stenting.

## Case History

A 61-year-old patient showed, in a few months, paresthesias of the scalp at occipital level, followed by local pains. Subsequently, intermittent diplopia appeared, then significant headaches with tingling of the lips on the left side, which motivated the patient to go to the emergency unit. Among his medical history, we noted a prostatic neoplasia treated by radiotherapy and hormonotherapy.

The ophthalmological work-up showed bilateral papilledema, intermittent visual blur, and horizontal binocular diplopia in evolution for 3–4 weeks, requiring an emergency focus by computed tomography (CT) scan.

**Figure 1:**
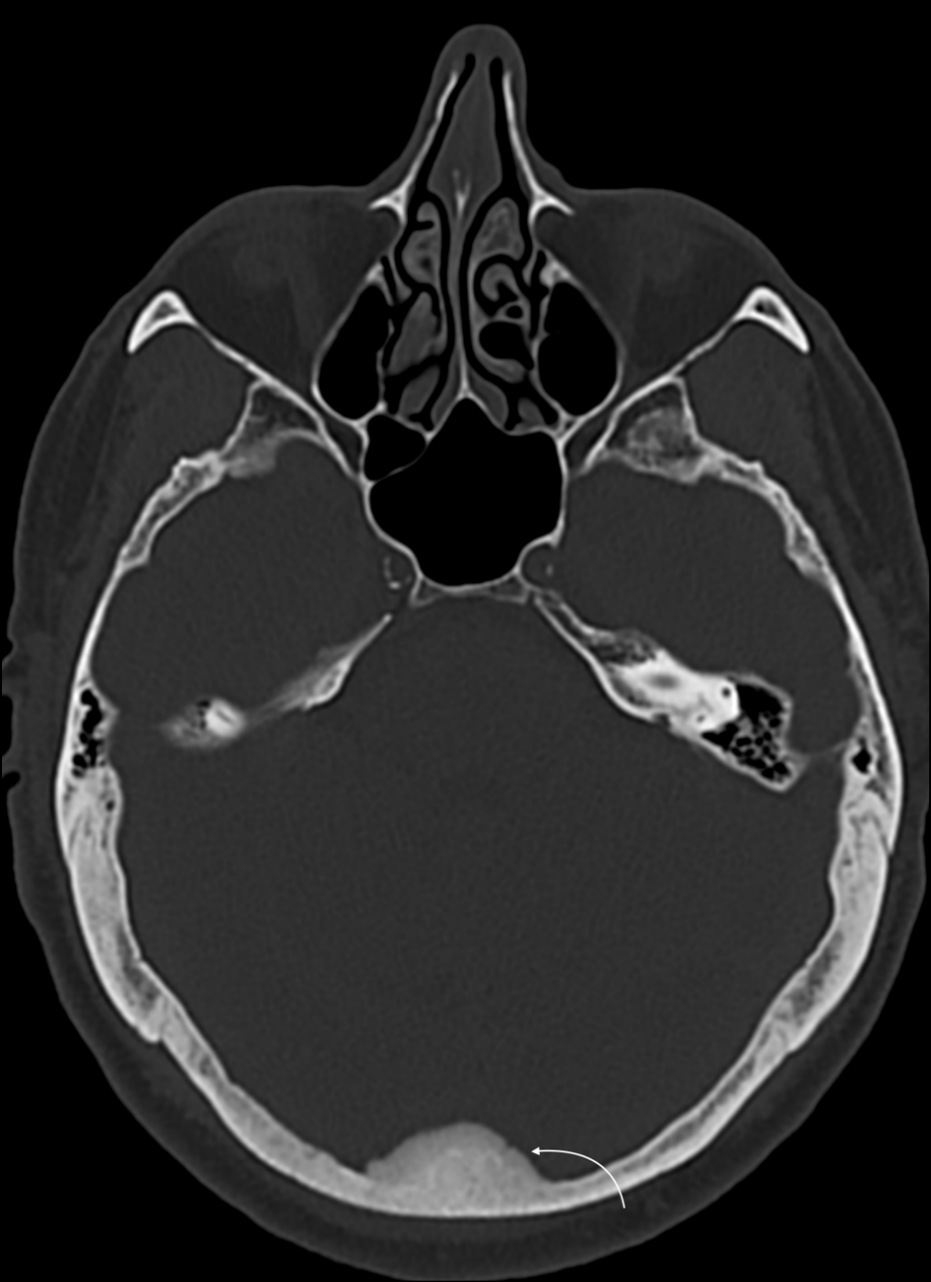


The non-enhanced cranial CT showed medial occipital sclerotic lesion at the level of the torcular, with periosteal reaction of the inner table, suggesting a bone metastasis (Figure 1, white arrow).

**Figure 2:**
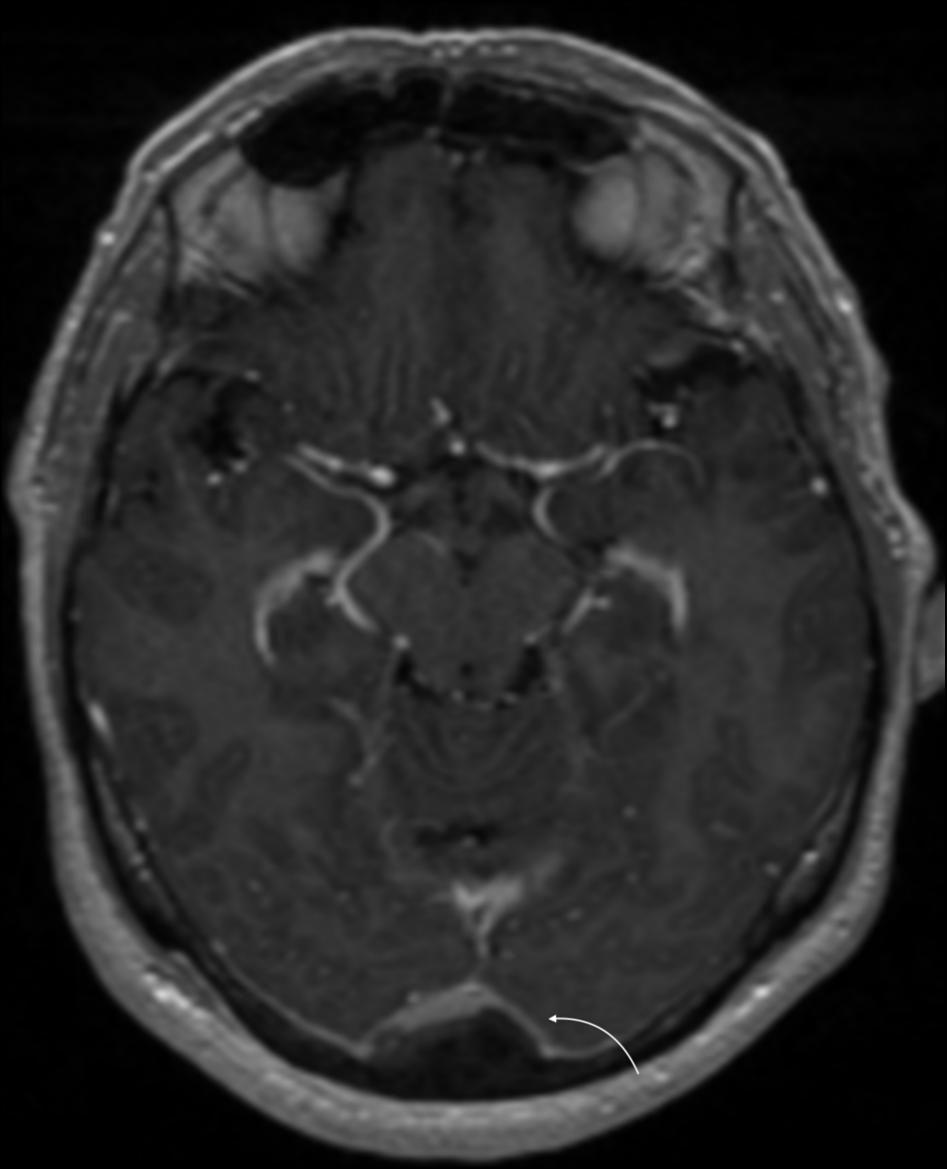


**Figure 3:**
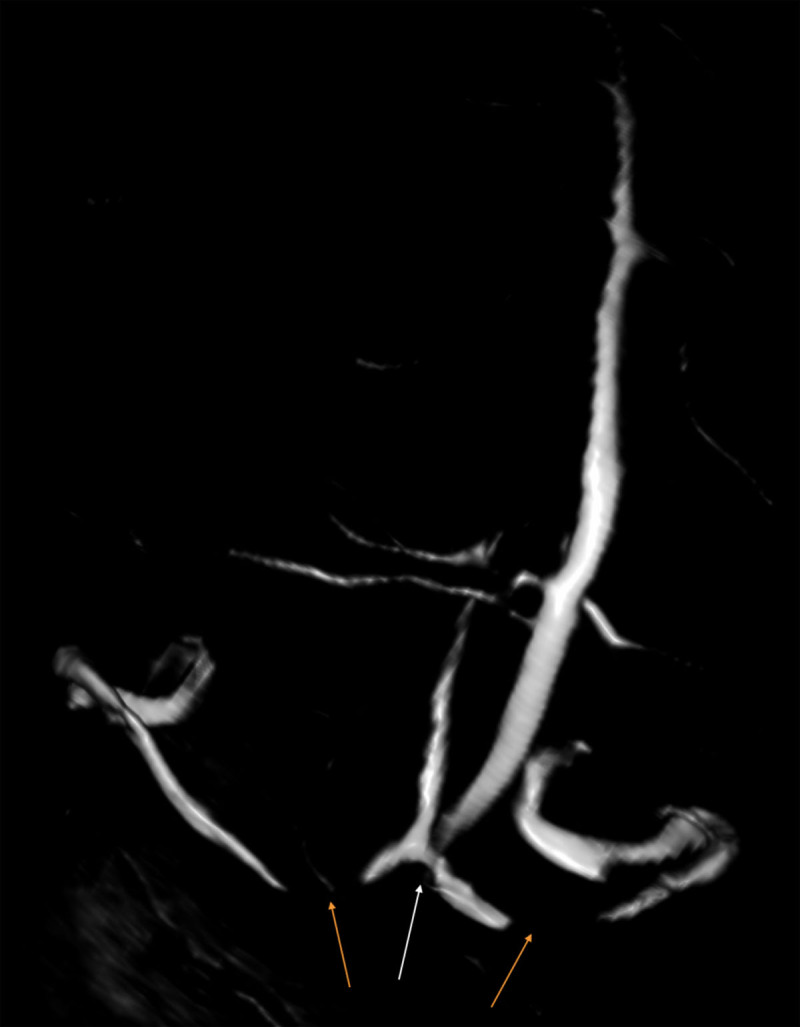


A complement by magnetic resonance venography comfirmed an enhanced skull sclerotic metastase, which occluded the torcular (Figures 2 and 3, white arrow) and the proximal portion of the two transverse sinuses without venous infarct (Figure 3, orange arrow), and showed intracranial hypertension signs, such as prominent subarachnoid space around the optic nerve, partial empty sella, and acquired tonsillar ectopia.

## Comment

Extrinsic stenosis of dural sinus is a rare cause of increased intracranial pressure (ICP). Sinus venosus thrombosis is the principal differential diagnosis in this sub-acute context, no matter what the origin (e.g., inflammatory disease or coagulations disorders). Most cases of dural sinus occlusion in oncologic context are due to thrombotic complications, not extrinsic stenosis.

Rare cases of tumoral compression of dural sinus are reported in literature, including neuroblastoma, plasmocytoma, breast and prostate carcinoma, or renal cell carcinoma.

The stenting of dural sinus has now been demonstrated to be a valid alternative to surgery in treatment of ICP, secondary to dural venus stenosis, especially sinus transvers stenting, which is a safe and clinically durable technique [1]. However, such new focal dural sinus stenosis close to stents, mainly in extrinsic stenosis. In our case, this treatment can be discussed, despite the topography of the stenosis. Finally, the ophtalmological follow-up shows favorable visual evolution with hormonotherapy, dexamethasone, and diamox. Thus, a non-invasive treatment are mostly indicated.
